# Changes in Sedentary Behaviours and Associations with Physical Activity through Retirement: A 6-Year Longitudinal Study

**DOI:** 10.1371/journal.pone.0106850

**Published:** 2014-09-26

**Authors:** Mehdi Menai, Léopold Fezeu, Hélène Charreire, Emmanuelle Kesse-Guyot, Mathilde Touvier, Chantal Simon, Christiane Weber, Valentina A. Andreeva, Serge Hercberg, Jean-Michel Oppert

**Affiliations:** 1 Université Paris 13, Sorbonne Paris Cité - EREN (Equipe de Recherche en Epidémiologie Nutritionnelle), U1153 Inserm, Inra, Cnam, Centre de Recherche en Epidémiologie et Biostatistiques; CRNH IdF, Bobigny, France; 2 Paris-Est Créteil University, Department of Geography, Lab-Urba, Urbanism Institute of Paris, Paris, France; 3 CARMEN, INSERM U1060/Université de Lyon 1/INRA U1235 Lyon, France; 4 Laboratoire Image, Ville et Environnement, Université de Strasbourg, Strasbourg, France; 5 Department of Public Health, Hôpital Avicenne (AP-HP), Bobigny, France; 6 Université Pierre et Marie Curie-Paris 6, Dept of Nutrition Pitié-Salpêtrière Hospital (AP-HP), Centre for Research on Human Nutrition Ile-de-France (CRNH IdF), Institute of Cardiometabolism and Nutrition (ICAN), Paris, France; Oregon Health & Science University, United States of America

## Abstract

Changes in sedentary behaviours and physical activity according to retirement status need to be better defined. Retirement is a critical life period that may influence a number of health behaviours. We assessed past-year sedentary behaviours (television, computer and reading time during leisure, occupational and domestic sitting time, in h/week) and physical activity (leisure, occupational and domestic, in h/week) over 6 years (2000–2001 and 2007) using the Modifiable Activity Questionnaire in 2,841 participants (mean age: 57.3±5.0 y) of the SU.VI.MAX (Supplementation with Antioxidants and Minerals) cohort. Analyses were performed according to retirement status. Subjects retired in 2001 and 2007 (40%) were those who spent most time in sedentary behaviour and in physical activity during and outside leisure (p<0.001). Leisure-time sedentary behaviours increased in all subjects during follow-up (p<0.001), but subjects who retired between 2001 and 2007 (31%) were those who reported the greatest changes (+8.4±0.42 h/week for a combined indicator of leisure-time sedentary behaviour). They also had the greatest increase in time spent in leisure-time physical activity (+2.5±0.2 h/week). In subjects not retired 2001 and 2007 (29%), changes in time spent watching television were found positively associated with an increase in occupational physical activity (p = 0.04) and negatively associated with changes in leisure-time physical activity (p = 0.02). No consistent association between changes in sedentary behaviours and changes in physical activity was observed in subjects retired in 2001 and 2007. Public health interventions should target retiring age populations not only to encourage physical activity but also to limit sedentary behaviours.

## Background

According to the World Health Organization, the number of people 60 years of age or older worldwide will grow from 600 million to 2 billion by 2050. A large range of determinants (sociological, medical and technological) alter the habits and the behaviours of active and retired populations. The transition to retirement is considered as a major life event in terms of financial as well as behavioural modification, including important changes in sedentary and physical activity behaviours [1].

There is growing interest in sedentary behaviour and related health outcomes in adults [2]. Sedentary behaviour refers to any waking behaviour characterised by an energy expenditure ≤1.5 METs while in a sitting or reclining posture [3] (a MET or Metabolic Equivalent Task is the ratio of the working metabolic rate of an activity divided by the resting metabolic rate [4]). This includes sitting and watching television (TV), along with other forms of screen-based entertainment [5]. Recently, sedentary behaviour was shown to be associated with increased risk of type 2 diabetes [6], [7], cardiovascular disease [6], [7], metabolic syndrome [8] and all-cause mortality [6], [9], independent of habitual physical activity levels. The transition to retirement has been associated with an increase in TV viewing time in only three previous reports [10]–[12], but there is no evidence for the influence on other sedentary behaviours, such as computer time, reading time or overall sitting time.

In addition to the recognised health benefits of physical activity at all ages, recent studies point to particularly favourable effects in aging subjects. In elderly populations, physical activity was recently shown to be inversely associated with risk of mortality [13], dementia [14], some types of cancer [15], and depression [16]. However, most previous studies have focused on leisure-time physical activity [12], [17]–[21] and only few studies have taken into account domestic and occupational physical activity [20], [21].

In a recent systematic review, half of the included studies reported a negative association between physical activity and TV time, no relation was found with computer time, and there was no mention of reading time [22]. Incongruity across study results could reflect differences in methodology as well as in socio-demographic indicators. Considering the protective effect on health of physical activity and the potentially harmful effects of sedentary behaviour [23], defining at-risk populations or stages of life where physical activity and specific sedentary behaviours are related could help fine-tune public health policies targeting both exposures simultaneously. At present, the relationships between sedentary behaviour and physical activity in aging populations remain poorly documented, not permitting the assessment of the association before, during and after retirement [22].

The objectives of the present study, which used a longitudinal design in a sample of middle-aged French adults, were 1) to describe the 6-year changes in different types of sedentary behaviours and different domains of physical activity according to retirement status, and 2) to investigate the relationships between changes in sedentary behaviours and changes in physical activity according to retirement status.

## Methods

### Ethics statement

Subjects provided written informed consent to the study which was conducted according to guidelines laid down in the Declaration of Helsinki and was approved by the Ethics Committee for Studies with Human Subjects at Paris-Cochin Hospital (CCPPRB n° 706 and n° 2364, respectively) and the Commission Nationale de l’Informatique et des Libertés (CNIL n° 334641 and n° 907094, respectively).

### Subjects

Subjects were participants in the “Supplémentation en VItamines et Minéraux AntioXydants” (SU.VI.MAX) cohort. The design, methods and rationale of the SU.VI.MAX study have been described elsewhere [24]. It was initially designed as a randomised, double-blind, placebo- controlled primary prevention trial to test the efficacy of daily supplementation with antioxidant vitamins and minerals at nutritional doses in reducing the incidence of ischaemic heart disease, cancer and overall mortality [25]. Following a 5-month national multimedia campaign that included TV, radio and newspapers, volunteer subjects, not selected for any specific risk factors, were included in 1994–1995 for a planned follow-up of 8 years (men: 45–60 y, women: 35–60 y). Each subject underwent a yearly visit alternating between a clinical examination and biological sampling. From the full SU.VI.MAX cohort (N = 12,741), a total of 6,850 subjects who had agreed to participate in a post-supplementation observational follow-up were recruited for the SU.VI.MAX 2 study (2007–2009).

### Physical activity and sedentary behaviours

Physical activity and sedentary behaviours were assessed in 2001 and 2007 using a French self-administered version [26] of the Modifiable Activity Questionnaire (MAQ) [27]. This instrument assesses past 12-month physical activity in various domains of everyday life. Physical activity assessment using the MAQ has been validated against energy expenditure measurements using the double-labelled water technique, and the test-retest properties of the questionnaire have been shown [28]. The questionnaire has been described in detail elsewhere [27]. Briefly, subjects were asked to report all leisure-time physical activity performed at least 10 times for 10 min per session over the past 12 months. Detailed information was collected concerning the type of leisure-time activity (walking, cycling, swimming, gardening, etc.). The frequency and duration of each activity was reported. Leisure-time activities were classified according to their intensity, based on their estimated metabolic cost, as moderate (3–6 METs) and vigorous (>6 METs) activity. Thus, physical activity variables used in the analyses included moderate and vigorous leisure-time physical activity, selected physical activities (e.g. walking, gardening, etc.), and the sum of the overall time spent in leisure-time physical activity.

The questionnaire included items about the time usually spent (in hours and minutes per day) at home watching TV/video, using a computer or playing video games, or reading for leisure. Time spent in each leisure sedentary occupation and the sum of all time periods spent in these leisure sedentary occupations were used in the analyses.

Assessment of occupational physical activity was based on the number of hours during which an individual participated in physically demanding activities during an average work day, for each job held over the past year. Occupational sedentary behaviour was based on the number of hours sitting during an average work day, for each job held over the past year. The original version of the MAQ only explored physical activity during leisure-time and at work. To also take into account physical activity unrelated to occupation or leisure, subjects were separately asked to report any domestic physical activity which included activities related to housekeeping, use of active transportation to run errands, etc. that had been performed at least 10 times for 10 min each session over the past 12 months. These questions were asked in the same format as in the occupational section of the questionnaire, including sitting.

### Sociodemographic covariables

Retirement status was assessed by self-report during the 2001 (baseline for the present study) and 2007 (follow-up) visits. The population was divided into 3 categories, according to their baseline and follow-up retirement status: 1) subjects who were not retired in 2001 and 2007, 2) subjects who retired between 2001 and 2007, and 3) subjects who were retired in 2001 and 2007. Sex, date of birth and educational level were assessed at entry using a self-administered questionnaire. Level of education was coded into three categories according to the highest certification obtained (primary school, high school, university or equivalent). Smoking status (never, former, current) was assessed in September 1998 by a separate questionnaire sent to the entire cohort. Height and weight were measured during the 2001 and 2007 visits. BMI was calculated as body weight (in kilograms) divided by the squared height (kg/m^2^).

### Statistical analyses

For each subject, changes between 2001 and 2007 in indicators of sedentary behaviour and physical activity were computed as the value recorded in 2007 minus the value recorded in 2001. Continuous variables were summarized by calculating the mean ± standard deviation (SD). We used one-way ANOVA to assess differences in continuous variables across retirement groups, with a Bonferroni correction applied for multiple comparisons. T-tests were used to assess post-hoc differences between retirement groups and to assess changes in the continuous variables during follow-up. We used chi-square tests to assess differences in categorical variables across retirement groups. Associations between changes in sedentary behaviours and changes in physical activity according to retirement status evolution between 2001 and 2007 were assessed using a multivariate generalised linear model. Covariates included sex, age in 2001, educational level, smoking status and occupational physical activity at baseline when appropriate. For all analyses, the significance level was set at 0.05 and all tests were two-sided. All statistical analyses were performed using SAS software (version 9.3, SAS Institute Inc., Cary, NC, USA).

## Results

### Comparisons between subjects included and not included in the analyses

Among subjects initially included in the SU.VI.MAX study, we focused the present analyses on subjects with available data from the physical activity questionnaires both in 2001 and 2007 (n = 3,458 subjects available), aged 45 years or older at entry into the study (in order to have a similar age range in both genders) (n = 3,006 subjects). In addition, subjects who reported being confined to bed more than 4 weeks in the pastyear before completing the questionnaires were excluded (n = 165), thus obtaining a final sample of n = 2,841 (1,453 men and 1,388 women).

Compared to subjects examined in 2007 (SU.VI.MAX 2 study) but not included in the present analyses, our study population comprised more men (51.1 vs. 36.2%, p<0.001), more subjects with a university education level (42.9 vs. 39.5%, p<0.001), and they had a lower mean BMI (24.3 kg/m^2^ vs. 24.5 kg/m^2^, p = 0.03), and were slightly older at baseline (57.0 y vs. 55.2 y, p<0.001).

### Baseline characteristics of the study population

Twenty nine percent of subjects were not retired in 2001 and 2007, 31.4% were retired between 2001 and 2007, and 39.6% of the sample were retired in 2001 and 2007 (Table 1). Subjects retired in 2001 and 2007 were mostly men, older and less educated than their employed counterparts (Table 1). At baseline, subjects retired in 2001 and 2007 had higher total levels of leisure-time sedentary behaviour, spent more time watching TV and reading, and spent twice the time in overall leisure-time physical activity (Table 2) compared to other two groups. These subjects also engaged in twice as much walking and gardening.

**Table 1:** Characteristics of the study population according to retirement status.

	Not retired in2001 and 2007	Retirement between2001 and 2007	Retired in 2001and 2007	p
N	1126	891	824	
Sex (% men)	38.3	55.6	64.0	<0.001
Age (years)	53.1 (3.2)	57.1 (3.6)	62.3 (3.3)	<0.001
BMI (kg/m^2^)	24.1 (3.5)	24.9 (3.5)	25.4 (3.4)	<0.001
*Educational level*				
Primary school	14.5	17.3	22.9	<0.001
High school	33.4	39.7	46.6	
University orequivalent	52.1	43	30.4	
*Smoking status*				
Never smoker	50.9	50.8	49.3	0.075
Former smoker	37.6	37.3	42.1	
Current smoker	11.5	11.9	8.6	

Values are mean (SD) or %.

p values are from ANOVA or Chi-square tests.

**Table 2:** Sedentary behaviours and physical activity at baseline (2001) and changes during 6-year follow-up.

	Baseline				Change			
	Not retired in 2001 and 2007	Retirement between 2001 and 2007	Retired in 2001 and 2007	p	Not retired in 2001 and 2007†	Retirement between 2001 and 2007††	Retired in 2001 and 2007†††	p
n	1126	891	824		1126	891	824	
*Sedentary behaviours (h/week)*								
Total leisure sedentary behaviour	21.8 (10.7)	23.3 (11.4)	28.9 (12.1)	<0.001^b^^,^ c	4.7 (0.35)	8.4 (0.42)	4.2 (0.41)	<0.001^a^^,^ b
Television viewing during leisure	12.8 (8.2)	13.9 (8.0)	17.9 (9.2)	<0.001^a^^,^ b ^,^ c	1.5 (0.19)	3.0 (0.24)	0.9 (0.27)	<0.001^a^^,^ b
Computer use during leisure	2.1 (4.0)	2.3 (4.5)	2.6 (5.0)	0.047^c^	2.5 (0.17)	4.1 (0.24)	2.8 (0.21)	<0.001^a^^,^ b
Reading during leisure	7.1 (5)	7.1 (5.3)	8.5 (5.6)	0.017^b^^,^ c	0.6 (0.19)	1.3 (0.22)	0.4 (0.20)	0.006^b^
Occupational sitting	16.9 (13.3)	14.2 (13.0)	-	<0.001^a^	−1.7 (0.31)	−14.2 (0.45)	-	<0.001^a^
Domestic sitting	1.8 (4.9)	2.3 (8.0)	4.8 (9.9)	<0.001^b^^,^ c	0.3 (0.26)	1.81 (0.42)	−1.1 (0.53)	<0.001^b^^,^ c
*Physical activity (h/week)*								
Total leisure	3.4 (3.9)	3.6 (4.2)	6.7 (6.6)	<0.001^b^^,^ c	0.7 (0.11)	2.5 (0.18)	−0.6 (0.23)	<0.001^a^^,^ b ^,^ c
Moderate leisure	2.7 (3.6)	3.0 (3.9)	5.7 (6.3)	<0.001^b^^,^ c	0.3 (0.11)	1.7 (0.17)	−0.7 (0.22)	<0.001^a^^,^ b ^,^ c
Vigorous leisure	0.6 (1.5)	0.6 (1.4)	0.9 (2.1)	0.045^b^^,^ c	0.1 (0.06)	0.4 (0.06)	−0.1 (0.07)	<0.001^a^^,^ b
Walking	0.8 (1.5)	0.9 (1.7)	1.6 (2.2)	<0.001^b^^,^ c	0.3 (0.08)	0.9 (0.09)	0.1 (0.09)	<0.001^a^^,^ b
Gardening	0.7 (1.7)	0.9 (1.9)	2.0 (3.7)	<0.001^b^^,^ c	0.2 (0.05)	0.7 (0.09)	−0.1 (0.13)	<0.001^a^^,^ b
Swimming	0.1 (0.3)	0.1 (0.3)	0.1 (0.4)	0.50	0.006 (0.01)	0.04 (0.01)	−0.03 (0.01)	0.002^b^
Biking	0.2 (0.6)	0.2 (0.7)	0.3 (1.1)	0.043^b^^,^ c	0.04 (0.02)	0.1 (0.05)	−0.05 (0.03)	0.01^b^
Occupational	14.1 (13.5)	14.1 (13.3)	-	0.78	−3.4 (0.56)	−14.1 (0.46)	-	<0.001^a^
Domestic	7.6 (8.5)	6.6 (7.3)	9.1 (10.0)	<0.001^b^^,^ c	−0.7 (0.33)	3.6 (0.49)	−0.1 (0.67)	<0.001^a^^,^ b

Values are mean (SD) for baseline and mean (SE) for changes. p values are from ANOVA with adjustment on sex and age.

a Comparisons between subjects not retired in 2001 and 2007 and subjects who retired between 2001 and 2007 are significantly different (p<0.05).

b Comparisons between subjects who retired between 2001 and 2007 and subjects retired in 2001 and 2007 are significantly different (p<0.05).

c Comparisons between subjects not retired in 2001 and 2007 and subjects retired in 2001 and 2007 are significantly different (p<0.05).

† Except for vigorous leisure time physical activity and swimming, all values for changes are significantly different from 0.

†† All values for changes are significantly different from 0.

††† Except for reading, domestic physical activity, vigorous leisure physical activity, walking, gardening and biking, all values for changes are significantly different from 0.

### Six-year changes in sedentary behaviours and physical activity

Between 2001 and 2007, total leisure-time sedentary behaviour, TV viewing and computer use increased in all three groups (p<0.001 in each group for the comparison between follow-up and baseline levels) (Table 2). For subjects retired between 2001 and 2007, there was an increase in both leisure-time and domestic physical activity (all p<0.01, data not shown). For subjects retired in 2001 and 2007, time spent in total leisure-time physical activity and moderate-intensity leisure-time physical activity decreased (p<0.005, data not shown).

Between 2001 and 2007, total leisure-time sedentary behaviour, TV viewing and computer use increased to a larger extent in subjects retired between 2001 and 2007 compared to the two other groups (Table 2). Leisure-time physical activities, except for swimming and biking also increased more in subjects retired between 2001 and 2007 compared to the two other groups (Table 2). When comparing subjects not retired in 2001 and 2007 and subjects retired in 2001 and 2007, there was no significant difference for changes in sedentary behaviours during leisure. In contrast, changes in total and moderate-intensity leisure-time physical activities were significantly higher for subjects not retired in 2001 and 2007 compared to subjects retired in 2001 and 2007 (Table 2).

The associations of retirement with sedentary behaviours and physical activity shown in Table 2 are consistent with models adjusted for age, sex, educational level, smoking status, and initial sedentary and physical activity behaviour levels (data shown in Table S1).

### Associations between changes in sedentary behaviour and changes in physical activity

In subjects not retired in 2001 and 2007, changes in time spent TV viewing were positively related to changes in occupational physical activity (p = 0.04) (Table 3). In the same group, changes in time spent in total and moderate-intensity leisure-time physical activity was negatively associated with changes in time spent watching TV (p = 0.02 and p = 0.02 respectively). No association was found for subjects retired between 2001 and 2007 or for subjects retired in 2001 and 2007 (data not shown).

**Table 3:** Relations between changes in time spent in sedentary behaviours (dependent variables) and changes in physical activity (exposure variables) during the 6-year follow-up for subjects working in 2001 and 2007.

	Television viewing during leisure		Computer use during leisure		Reading during leisure		Occupational sitting		Domestic sitting	
Physical activity (h/week)	β	p	β	p	β	p	β	p	β	p
Total leisure	−**0.04**	***0.02***	0.01	*0.50*	−0.01	*0.66*	−0.04	*0.52*	−0.11	*0.22*
Moderate leisure	−**0.04**	***0.02***	0.02	*0.31*	0.03	*0.23*	−0.07	*0.27*	−0.12	*0.18*
Vigorous leisure	−0.04	*0.10*	0.01	*0.69*	−0.03	*0.39*	−0.10	*0.38*	−0.19	*0.31*
Occupational	**0.02**	***0.04***	0.002	*0.82*	0.02	*0.13*	−0.03	*0.53*	−0.01	*0.87*
Domestic	0.08	*0.02*	0.01	*0.7*	−0.01	*0.84*	**0.20**	***0.001***	**0.05**	***0.046***

Beta coefficients are from linear regression analyses with each change in sedentary behaviour as outcome variable. Models were adjusted for age, sex, educational level, smoking status, physical activity at baseline and baseline values of the respective outcome variable.

## Discussion

In this study in French subjects, we studied the 6-year changes in different types of sedentary behaviour and different domains of physical activity according to retirement status, and we investigated the relationships between changes in sedentary behaviours and changes in physical activity according to retirement status. All subjects increased their sedentary behaviour during follow-up, but those retired between 2001 and 2007 showed the greatest changes. They also had the greatest increase in time spent in leisure-time physical activity. Subjects retired in 2001 and 2007 were those who spent the most time in sedentary behaviour, leisure-time physical activity and domestic leisure-time physical activity. Finally, leisure-time physical activity was inversely associated with time spent TV viewing in subjects not retired in 2001 and 2007. These findings extend those of our previous report documenting changes in sedentary behaviour and physical activity over three years in subjects from the same population [12].

In subjects retired between 2001 and 2007, it was noticeable that the mean increase in total sedentary behaviour was about three times higher (+8.4 h/week) than the mean increase in leisure-time physical activity (+2.5 h/week). The greatest change in sedentary behaviour during leisure-time was for computer use compared to reading and TV viewing. This behavioural change appears in line with the rapidly expanding use of computers and the Internet across different population groups including aging subjects, during these same years (2001–2007) [29]. It underscores the importance of assessing different dimensions of sedentary behaviours and not only TV viewing, known to increase through retirement [11], [12].

In addition to sedentary behaviours, this study is one of the few that performed a detailed assessment of changes in different types of leisure-time physical activities in subjects transitioning to retirement [10]. In our subjects, the increase in moderate-intensity activity (such as walking and gardening) accounted on average for about two thirds of the increase in total leisure-time physical activity. This is in agreement with results from previous studies where transition to retirement was associated with an increased sports participation [11], with increased total leisure-time physical activity score [10], [18] and with an increased proportion of subjects meeting physical activity recommendations [17].

There are several potential explanations for the increase in leisure-time physical activity with retirement. First, retirement leads to a decrease in time constraints possibly corresponding to more free-time available. Second, individuals likely become increasingly health-conscious in the context of aging, which could increase motivation to engage in more recreational physical activity. Finally, increased leisure-time physical activity after retirement may provide a new daily routine and new opportunities for social interactions [30].

For subjects not retired in 2001 and 2007 the changes in time spent watching TV during leisure-time were differentially associated with changes in time spent being active at work (positive association) and time spent being active during leisure (negative association). The positive association with changes in active occupational time may intuitively be interpreted as compensatory behaviour and the need for rest outside work. The negative association with changes in active leisure-time might reflect choices made in terms of budget time in this working population. Although there is no previous study reporting such associations in a longitudinal design, negative cross-sectional associations were shown between TV time and leisure-time physical activity [31], [32]. The lack of significant results for subjects retired between 2001 and 2007 and those retired in 2001 and 2007 can be interpreted as subjects having more availability after retirement and consequently not to make choices between leisure-time sedentary behaviour and physical activity.

Some limitations of this study need to be mentioned. First, measurements of physical activity and sedentary behaviours were derived from self-reporting, which can cause misclassification bias mostly because of over-reporting of physical activity [33]. However, there is no reason to expect that retirement status would have repercussions on that misclassification. In addition, the applicability of the MAQ to older subjects may be discussed. Regarding reliability, in the first description of the MAQ by Kriska et al. [27], 1–3 week test-retest correlations for past-year leisure-time physical activity were found to be of about the same magnitude in the older compared to the younger adult subjects (37–59 y, rho = 0.88 and 21–36 y, rho = 0.92, respectively). Second, we had information about the employment status (retired or not) at the time of data collection (2001 and 2007) but the actual date of retirement amongst these subjects and the reasons for retirement were not available. A possible selection bias could occur if some participants had retired earlier because of poor health, which might have negatively impacted their physical activity. To prevent, at least in part this limitation, subjects who reported being confined to bed more than 4 weeks in the past year before completing the questionnaires were excluded from the analyses. In addition, smoking habits were self-reported two years before the first wave of physical activity assessment, leading to a potential misclassification bias regarding subjects who had quit smoking between the two assessment periods. Third, despite the retirement age in our population (58–64 y) being consistent with the retirement age in France at the time of the study, our subjects were volunteers participating in a nutritional intervention study. Therefore, they generally had a higher educational levels and occupational status, along with a healthier lifestyle than the general population [25]. For these reasons extrapolation of these findings must be done cautiously.

## Conclusions

In subjects transitioning to retirement, both sedentary behaviour and physical activity during leisure-time increased substantially over the 6-year follow-up period. However, the mean increase in sedentary behaviour during leisure-time was about three times higher than the mean increase in leisure-time physical activity, with the greatest change seen in computer use compared to reading and TV viewing. This may be of serious concern considering the growing evidence of the deleterious health effects of sedentary behaviours. This emphasizes the need to test interventions and to reinforce public health measures aimed at decreasing sedentary behaviours and increasing regular physical activity during the critical retirement period.

## Supporting Information

Table S1 **Relations between changes in time spent in sedentary behaviours or changes in physical activity with retirement status during the 6-year follow-up (linear regression analysis.**
(DOC)
