# Supplementary material for: Changes in Sedentary Behaviours and Associations with Physical Activity through Retirement: A 6-Year Longitudinal Study
Source: PLoS One. 2014 Sep 26;9(9):e106850. doi: 10.1371/journal.pone.0106850 (PMC4178017; doi:10.1371/journal.pone.0106850)
Supplement: Table S1 — Relations between changes in time spent in sedentary behaviours or changes in physical activity with retirement status during the 6-year follow-up (linear regression analysis. (DOC) [file pone.0106850.s001.doc]

Table S1 - Relations between changes in time spent in sedentary behaviours or changes in physical activity with retirement status during the 6-year follow-up (linear regression analysis)

|  | Not retired in 2001 and 2007 (reference) | Retiring between 2001 and 2007 | | Retired in 2001 and 2007 | |
| --- | --- | --- | --- | --- | --- |
| *Sedentary behaviours* |  | β | p | β | p |
| Total leisure sedentary behaviour | - | 3.61 | <0.001 | 0.99 | 0.23 |
|  |  |  |  |  |  |
| Television viewing during leisure | - | 1.43 | <0.001 | -0.10 | 0.84 |
|  |  |  |  |  |  |
| Computer use during leisure | - | 1.65 | <0.001 | 0.59 | 0.18 |
|  |  |  |  |  |  |
| Reading during leisure | - | 0.48 | 0.11 | -0.06 | 0.89 |
|  |  |  |  |  |  |
| Occupational sitting | - | -14.24 | <0.001 |  |  |
|  |  |  |  |  |  |
| Domestic sitting | - | 1.21 | 0.01 | 0.40 | 0.53 |
|  |  |  |  |  |  |
| *Physical activity* |  |  |  |  |  |
| Total leisure | - | 2.08 | <0.001 | 0.89 | 0.009 |
|  |  |  |  |  |  |
| Moderate leisure | - | 1.65 | <0.001 | 0.95 | 0.002 |
|  |  |  |  |  |  |
| Vigorous leisure | - | 0.18 | 0.06 | -0.15 | 0.24 |
|  |  |  |  |  |  |
| Occupational | - | -10.47 | <0.001 |  |  |
|  |  |  |  |  |  |
| Domestic | - | 4.57 | <0.001 | 3.26 | 0.0002 |

Beta coefficients are from linear regression analyses with each change in sedentary behaviour or change in physical activity as outcome variable and retirement status as primary explanatory variable modeled in 3 categories. Models were adjusted for age, sex, educational level, smoking status and baseline value of the respective outcome studied.
